# Adapting a workplace tobacco control program in small and medium-sized enterprises: identifying **“**key forms**”** for fidelity and flexibility using FRAME-IS

**DOI:** 10.3389/frhs.2026.1730791

**Published:** 2026-02-11

**Authors:** Miyuki Odawara, Junko Saito, Taichi Shimazu

**Affiliations:** 1 Division of Behavioral Sciences, National Cancer Center Institute for Cancer Control, National Cancer Center, Chuo-ku, Tokyo, Japan; 2 Teikyo University Graduate School of Public Health, Itabashi-ku, Tokyo, Japan

**Keywords:** adaptation, fidelity, FRAME-IS, implementation strategy, tobacco control, workplace healthpromotion

## Abstract

**Background:**

Workplace smoking cessation programs remain underused in small and medium-sized enterprises (SMEs) due to limited resources and implementation barriers. interactive assistance via eHealth for SMEs' employers and healthcare manager teams on tobacco control (eSMART-TC), was developed to address this gap. Effective contextual adaptation is essential for optimizing implementation strategies in real-world settings. To address this, we analyzed how the eSMART-TC strategies were adapted to diverse workplace contexts, aiming to clarify which components were essential and which could be modified to maintain effectiveness. In doing so, we proposed and operationalized the concept of “Key forms”—strategy elements that should remain unchanged to ensure fidelity. This study applied the Framework for Reporting Adaptations and Modifications to Evidence-based Implementation Strategies (FRAME-IS) to systematically document and analyze these adaptations.

**Methods:**

This study analyzed data from the waitlist control group of the eSMART-TC cluster randomized controlled trial. Program delivery to the waitlist control group was designed to permit context-specific adaptations, with the condition that all adaptations be documented using the FRAME-IS framework. Data sources included provider checklists, discussion transcripts, employee surveys, and company-level evaluations. Adaptations were assessed using the Function and Forms Matrix to distinguish core functions from specific forms and to identify modifications necessary for optimization.

**Results:**

Of 26 identified forms supporting four core functions, 19 were classified as essential for maintaining fidelity. Seven forms, primarily involving session frequency and duration, were modified during implementation. These reactive yet context-sensitive changes aimed to meet organizational needs and improve feasibility. Provider discussions facilitated the identification of acceptable adaptations, underscoring the value of co-creation.

**Conclusion:**

The FRAME-IS supported systematic documentation of reactive modifications and clarified which implementation strategy elements were essential vs. flexible. Defining key forms offers practical guidance for balancing fidelity and adaptation. Co-creation with providers was critical for optimizing implementation in resource-constrained workplace settings.

**Clinical Trial Registration:** The study protocol has been registered in the UMIN Clinical Trials Registry (UMIN-CTR; ID: UMIN000044526). Registered on 06/14/2021.

## 1 Introduction

Smoking imposes significant health and economic burdens on individuals, workplaces, and healthcare systems. It remains a major global public health issue, increasing the risk of various non-communicable diseases (NCDs) such as cancer, cardiovascular disease, and chronic respiratory conditions, and is a leading cause of premature mortality (1). Studies have demonstrated that smoking raises healthcare costs and reduces workplace productivity through increased absenteeism and disease burden (2–4). Despite this evidence, workplace tobacco control measures are inadequately implemented, particularly in small- and medium-sized enterprises (SMEs), where limited resources hinder adoption (5, 6). A study conducted in Japan reported that only 20% of Japanese SMEs engage in health promotion activities (7), and national surveys have shown lower implementation rates of comprehensive tobacco control measures in SMEs compared to large enterprises (8).

To address this gap, eSMART-TC, an interactive eHealth-based intervention, was developed to support SME employers and healthcare manager (HCM) teams in establishing and strengthening workplace tobacco control practices (9). A cluster randomized controlled trial demonstrated its effectiveness in increasing the implementation of tobacco control strategies and reducing smoking prevalence (10). The program includes 10–12 interview-based sessions over 6 months and employs three strategies developed via Implementation Mapping (11). By targeting management and HCMs, the program enables SMEs to sustain health promotion activities independently after external support ends. A key strength of eSMART-TC is its capacity to enhance health promotion in resource-limited SMEs. Since eSMART-TC was developed in collaboration with the Japan Health Insurance Association (JHIA), the largest health insurance provider for SMEs in Japan, its implementation is currently underway within JHIA-affiliated organizations.

Despite its potential, eSMART-TC faces practical challenges related to time demands and provider capacity. In the current implementation, the total time commitment is substantial, with employers spending approximately 4–6 h in sessions and health managers committing 16.5–19 h over 6 months, including 6.5–9 h in interviews and 10 h for implementation activities (9). JHIA public health nurses (PHNs) reported that the required time and frequency of sessions posed a burden, creating a barrier to adoption. To improve adoption among JHIA and SMEs while minimizing implementation costs, scheduling flexibility is essential to maintain effectiveness without overburdening stakeholders.

To ensure effective implementation across diverse SMEs, the adaptation and refinement of implementation strategies is essential, given their multi-component structures and operation within complex and evolving organizational settings (12, 13). Adaptation—defined as a deliberate modification to improve contextual fit (14)—and its broader counterpart, modification, which includes both planned and *ad hoc* changes (15), are increasingly recognized as essential not only for evidence-based practices but also for implementation strategies themselves (15, 16). Implementation strategies must be tailored to specific contexts, including Japanese SMEs; however, consistent documentation and reporting of adaptations and modifications remain insufficient (15).

Distinguishing core functions from adaptable forms within implementation strategies is a valuable approach for guiding adaptation. Core functions denote the essential purposes of an intervention—the mechanisms that achieve change—whereas forms are the specific activities used to enact those functions and can be tailored to local contexts (17, 18). The Function and Forms Matrix (18) provides a practical framework for organizing strategies. It includes three elements: the motivating need or problem, standardized core functions, and customizable forms. Employing this matrix helps ensure that adaptations preserve the intervention's integrity while allowing necessary context-specific adjustments.

The Framework for Reporting Adaptations and Modifications to Evidence-based Implementation Strategies (FRAME-IS) offers a structured, flexible tool for systematically documenting modifications (15). It comprises core and supplementary modules to classify the types, nature, and objectives of adaptations. Because of its practicality, FRAME-IS also facilitates hypothesis testing regarding how modifications influence core components of implementation strategies.

Although adaptations are often required, unplanned or misaligned ones may reduce the effectiveness and fidelity of strategies. Prior research indicates that non-systematic adaptations—those lacking a clear rationale or compromising core functions—can negatively affect outcomes (19). Therefore, when adapting strategies within eSMART-TC, it is essential to define their core functions and forms clearly and ensure that adaptations maintain those functions while addressing contextual needs.

This study aims to optimize the implementation strategies of eSMART-TC by identifying adaptations that align with core functions. Specifically, the objectives are [1] to define core functions and forms using the Function and Forms Matrix (18), and [2] to identify modifications that support optimization and sustainment of the program using the FRAME-IS framework (14).

## 2 Methods

### 2.1 Study setting and participants

This study analyzed the post-trial implementation of eSMART-TC conducted in the waitlist control group of a cluster randomized controlled trial (cRCT). In the cRCT, the intervention was delivered to the waitlist control group after completion of the primary intervention period, allowing for the observation and documentation of context-specific adaptations under routine implementation conditions.

The post-trial intervention involved 16 SMEs affiliated with the JHIA, excluding companies that withdrew before intervention delivery (*n* = 2). Characteristics of the participating SMEs are summarized in Table 1. Before study initiation, participating employers and HCMs received explanations of the study protocol and provided written informed consent. The study protocol was approved by the Ethics Committees of the National Cancer Center, Japan.

**Table 1:** Characteristics of participating small and medium-sized enterprises (SMEs) (*n* = 16).

Characteristic	Control group
	(*n* = 16)
Company size (employees in the headquarters and branches on the premises of that location)	
31–50	7 (43.8%)
51–100	7 (43.8%)
101–200	1 (6.3%)
201–300	1 (6.3%)
Company size (overall employees)	
<100 employees	10 (62.5%)
≥100 employees	6 (37.5%)
Geographic area	
Hokkaido	2 (12.5%)
Tohoku	2 (12.5%)
Hokuriku/Koshinetsu	4 (25.0%)
Kanto	4 (25.0%)
Tokai	0
Kansai	0
Chugoku	0
Shikoku	2 (12.5%)
Kyushu	2 (12.5%)
Industry type	
Construction	2 (12.5%)
Electricity, gas, heat supply, and water	1 (6.3%)
Finance and insurance	0
Information and communications	2 (12.5%)
Living-related and personal services and amusement services	0
Manufacturing	1 (6.3%)
Medical, healthcare, and welfare	2 (12.5%)
Scientific research, professional and technical services	0
Services, n.e.c.	2 (12.5%)
Transport and postal services	2 (12.5%)
Wholesale and retail trade	4 (25.0%)

**Figure 1:**
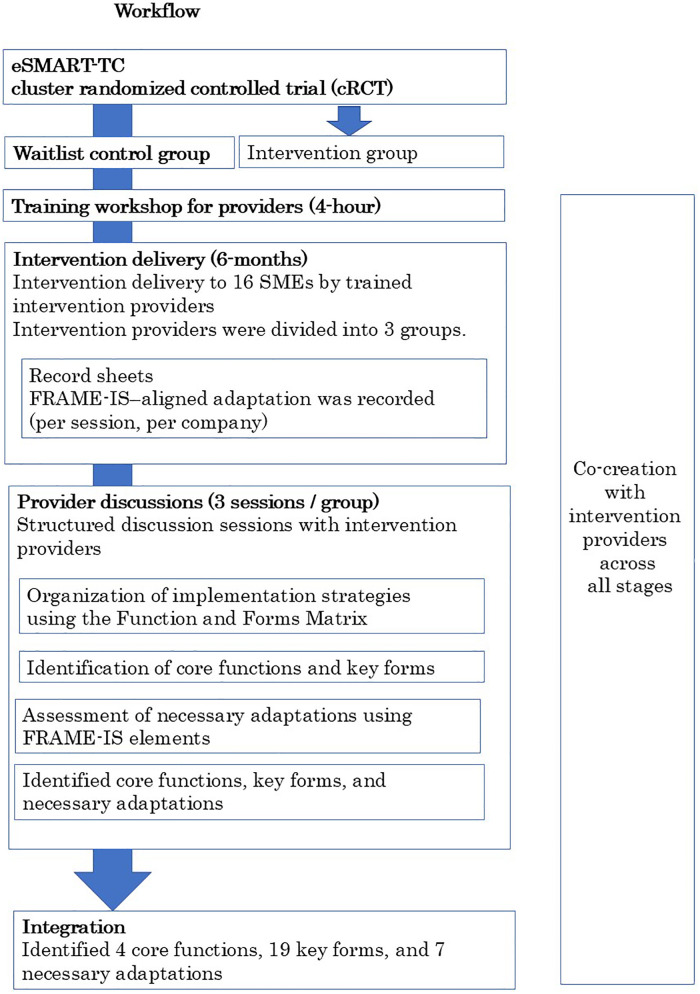
Overview of the study design and analytic workflow. The figure illustrates the relationship between the original eSMART-TC cluster randomized controlled trial and the post-trial implementation examined in this study. Following completion of the primary trial, the intervention was delivered to 16 SMEs in the waitlist control group after provider training. Implementation data were collected using FRAME-IS–aligned record sheets and structured provider discussion sessions. Analysis applied the Function and Forms Matrix to organize implementation strategies, identify core functions and key forms, and assess necessary adaptations. Co-creation with intervention providers occurred across all stages of the workflow.

Figure 1 illustrates the overall study design and analytic workflow, including post-trial implementation, data collection, and analysis.

#### 2.1.1 Provider sampling and participation

Intervention providers were selected based on completion of standardized training required to deliver the eSMART-TC implementation strategies. Eligibility criteria included participation in a 4-hour training workshop and submission of a written report demonstrating understanding of intervention delivery procedures and adaptation documentation using FRAME-IS. Twelve public health nurses (PHNs) affiliated with the Japan Health Insurance Association (JHIA) met these criteria. Of these PHNs, three did not deliver the intervention: one was assigned to the JHIA headquarters and therefore did not provide field-based intervention delivery; one was excluded due to job relocation during the study period; and one did not have any eligible SMEs assigned within their regional branch. To ensure adequate delivery capacity, three additional research team members (one physician and two PHNs) completed the same training and reporting requirements and served as intervention providers. Consequently, 12 providers delivered the intervention and participated in this study (Table 2). All 12 providers attended all scheduled discussion sessions and contributed to the documentation and evaluation of adaptations. Provider-company assignment: Each provider supported between one and four companies (median, 2; range, 1–4). Four providers supported one company each, three supported two companies, and one supported four companies. Two companies were supported by provider pairs working collaboratively. All 16 participating companies were represented through at least one provider in the discussion sessions.

**Table 2:** Demographic and professional characteristics of intervention providers.

Characteristic	PCPs	
	(*N* = 12)	
Characteristic	n	%
Sex		
Female	11	91.7
Male	1	8.3
Provider Type		
Public Health Nurse	10	83.3
Nurse Practitioner/Researcher	1	8.3
Medical Doctor/Researcher	1	8.3
1–10 years	6	50
>10 years	6	50

### 2.2 Structure and purpose of provider discussion sessions

Provider discussion sessions were conducted as structured, framework-based consensus discussions rather than open-ended focus groups. The primary purpose of these sessions was to review documented modifications, classify implementation strategies using the Function and Forms Matrix, and evaluate whether each modification constituted a necessary adaptation for optimization, based on FRAME-IS elements.

Intervention providers were divided into three groups based on the timing of intervention initiation, with each group comprising four providers. Each group participated in three discussion sessions, held after sessions 3 or 4, after sessions 6 or 7, and after the final intervention session. Each discussion lasted approximately 1–2 h, and all providers attended all scheduled sessions for their assigned group.

The discussions were facilitated by members of the research team using a structured guide informed by FRAME-IS modules and the Function and Forms Matrix. Providers reviewed their completed intervention checklists, shared experiences regarding implementation challenges, and jointly assessed the appropriateness and implications of the observed modifications. Session outputs were documented through detailed facilitator notes taken during discussions. Sessions were not audio-recorded, and these documented outputs were used as analytic inputs for subsequent qualitative analysis.

### 2.3 Operationalization of co-creation

In this study, co-creation was operationalized as a structured, iterative process through which intervention providers and the research team jointly reviewed, classified, and evaluated modifications to implementation strategies during and after intervention delivery, consistent with established co-creation methodologies in health and implementation research that emphasize collaborative knowledge generation through sustained researcher–practitioner partnerships (20).

The co-creation team comprised 12 intervention providers and 3 members of the research team (MO, JS, and TS), including the principal investigator and co-investigators with expertise in implementation science and workplace health promotion.

Co-creation was implemented through three sequential activities. First, intervention providers documented all deviations from planned implementation strategies using standardized checklists based on the FRAME-IS framework after each session. Second, these documented modifications were reviewed during structured provider discussion sessions, where implementation challenges were examined, and modifications were assessed in relation to optimization. Third, classifications of core functions and forms, identification of key forms, and determinations of necessary adaptations were finalized through consensus-based discussions informed by the Function and Forms Matrix and FRAME-IS elements.

Consensus was defined as agreement among all participating providers in each discussion session. Disagreements were resolved through facilitated discussion until all participants reached an agreement. All consensus decisions were documented in session notes and validated in subsequent sessions to ensure consistency across provider groups.

Intervention providers contributed information derived from implementation experience in workplace settings, and the research team facilitated the application of implementation frameworks during the review and classification process. Outputs from these co-creation activities informed the subsequent qualitative analysis.

### 2.4 Outcomes

The study assessed three primary outcomes. First, it identified the core functions and corresponding forms of eSMART-TC implementation strategies. Second, it determined the adaptations necessary to optimize these strategies. Finally, the program's effectiveness and acceptability were evaluated to contextualize adaptations. Effectiveness was measured by the smoking abstinence rate, while acceptability was assessed through participants' perceived effectiveness and satisfaction with the program.

### 2.5 Data collection

Data were obtained from (1) checklists completed by intervention providers, (2) employee surveys conducted at baseline and post-intervention, and (3) company surveys administered after the intervention in the waitlist control group of the eSMART-TC cRCT. Details about adaptations were collected using intervention checklists completed by providers for each session (Additional file 1).

The checklist listed the strategies to be delivered in sessions 1–10, consistent with the intervention group protocol. It included a section to record whether each strategy was executed. When a strategy was not implemented, this was considered a modification. Providers documented such changes in a designated section based on FRAME-IS modules 1–6: (1) description of the treatment and implementation strategies, (2) content modified, (3) nature of the modification, (4) rationale, (5) timing and whether planned, and (6) individuals involved in the decision. After each session, providers submitted the completed checklists to the research team.

Smoking status was measured through pre- and post-intervention employee surveys. Self-reported 7-day point-prevalence abstinence was assessed by calculating the percentage of current smokers at baseline who answered “yes” to the question in the post-intervention survey: “Have you smoked even one mouthful of tobacco [including all cigarettes (rolled, heated, and other types)] in the last seven days?” Program acceptability was evaluated based on HCMs' responses to the company survey regarding effectiveness (scale 1: not effective at all to 5: very effective) and satisfaction (scale 1: not satisfied at all to 5: very satisfied).

### 2.6 Data analysis

To address the study objectives, analysis was organized in two parts: (1) defining core functions and forms of the implementation strategies, and (2) identifying modifications that support optimization and potential sustainment of the program.

#### 2.6.1 Defining core functions and forms

A structured overview of the data analysis process is presented in Table 3, summarizing checklist review, group discussions, classification using the Function and Forms Matrix, and identification of key forms.

**Table 3:** Structured summary of data analysis steps including frameworks and methods.

Step	Title	Purpose	Framework or Method
Step 1	Data Collection & Validation	Document session implementation and adaptation	FRAME-IS modules
Step 2	Discussion with Intervention Provider	Identify core functions and necessary adaptations	Structured discussions
Step 3	Mapping Core Functions and Forms	Map implementation strategies using the Function and Forms Matrix	Function and Forms Matrix
Step 4	Identification of Key Forms	Determine forms critical for fidelity	Practical decision-making process
Step 5	Classification of Adaptations	Distinguish necessary adaptations	FRAME-IS
Step 6	Consensus Building and Validation	Enhance the validity and reliability of interpretation	Consensus discussions and cross-checking

The submitted checklists were reviewed by the author (MO), who requested corrections if any information was incomplete. Intervention providers were divided into three groups based on intervention start times. In each group, three discussion sessions lasting 1–2 h were held after sessions 3 or 4, sessions 6 or 7, and the final session. These discussions focused on evaluating whether modifications to the implementation strategies were appropriate, aiming to (1) identify core functions and forms and (2) determine necessary adaptations to optimize strategies for specific contexts and needs. To enhance trustworthiness, identified key forms and adaptations were confirmed through consensus discussions. Coding decisions were cross-checked by multiple authors to ensure consistency.

The Function and Forms Matrix (18) was used to identify core functions and forms. The research team first examined key challenges during implementation—such as limited executive engagement, low employee motivation to quit smoking, and difficulty sustaining tobacco control activities—and interpreted these as motivating needs the eSMART-TC intervention aimed to address. Based on these needs, four core functions were defined, each representing a central purpose of the implementation effort. Next, 26 implementation strategies were identified and categorized as forms—concrete, actionable components operationalizing their respective core functions. Each form was mapped to one of the four core functions according to the specific motivating need addressed. For example, strategies such as “setting goals for tobacco control” and “formally announcing the campaign to employees” were categorized as forms supporting the core function of “Ensuring Executive Engagement and Support”.

Classification was conducted through iterative discussions between the research team and intervention providers. The resulting structure, comprising motivating needs, core functions, and associated forms, is summarized in Table 4. To identify key forms, the submitted checklists were aggregated to examine the number of companies that modified each implementation strategy. Strategies not modified in any company were designated as key form candidate 1. Modified implementation strategies judged undesirable (e.g., forgotten, not executed by participants, or producing negative outcomes) were excluded from necessary adaptations for optimization. Implementation strategies modified under highly specific or non-generalizable circumstances, such as companies with smoking rates ≤5% or medical institutions with in-house cessation clinics, were also excluded. These were designated as key forms candidate 2. A consensus was reached with intervention providers, and key forms were formally identified.

**Table 4:** Function–forms matrix of eSMART-TC implementation strategies showing core functions, motivating needs, and Key forms.

No.	Core functions	Motivating need/problem	Forms	Key forms
1	Ensuring executive engagement and support	When executive involvement in health promotion is limited, awareness within the company remains low, and outcomes are less likely to be achieved.	The employer appoints an HCM responsible for smoking cessation measures and mandates the implementation of cessation initiatives as part of their duties.	✓
2			The employer participates in both the assessment and review sessions.	✓
3			The employer actively contributes to developing Work 1 (smoking cessation objectives and goals).	✓
4			The employer considers financial and non-financial resources in defining Work 2 (smoking cessation support content).	✓
5			The employer personally declares the smoking cessation measures to employees (in person, via web meeting, web platform, or printed materials).	✓
6			The employer participates in Sessions 1–3 (sessions on implementation planning).	
7	Ongoing facilitation tailored to the workplace context	The priority of health promotion often declines, requiring facilitation tailored to specific circumstances.	The HCM participates continuously in all sessions.	✓
8			The HCM completes and submits homework assignments (e.g., preparing materials).	✓
9			The HCM submits the action memo.	✓
10			The intervention provider informs the HCM about consultation availability via email/phone.	✓
11			The second to final sessions are conducted via the web and last for 30–60 min.	
12			Conduct 10–12 sessions over the course of 6 months.	
13	Supporting employees to quit smoking through the cessation campaign	Employees have low motivation to quit smoking.	The HCM introduces smokers to evidence-based smoking cessation methods, such as smoking cessation outpatient clinics and the use of nicotine patches or nicotine gum.	✓
14			The HCM prepares promotional materials for the smoking cessation campaign and creates a smoking cessation declaration form, then distributes them to all employees.	✓
15			The HCM provides focused smoking cessation support to individuals who are interested in quitting smoking.	✓
16			The HCM conducts sessions with smokers to assess their level of interest in quitting smoking, using face-to-face meetings, email, or web tools, and provides necessary advice accordingly.	✓
17			The HCM conducts internal announcements using multiple methods, including paper postings, web postings, morning meetings, and emails.	✓
18			The HCM conducts sessions with smokers or those interested in quitting (approximately once a week to once a month) to check their smoking cessation status through in-person, email, or web tools and provides necessary advice.	✓
19			Is a preparation period of 1 month acceptable?	
20			The campaign recruitment period is 1 month.	
21			The smoking cessation challenge period is 4 months.	
22			The entry method requires the submission of a smoking cessation declaration.	
23	Review and evaluation of the campaign	The PDSA cycle is not functioning, making sustained health promotion difficult.	The HCM arranges a review session within the company.	✓
24			The HCM conducts a review session within the company and sets goals for the next 6 months.	✓
25			The intervention provider evaluates the smoking control measures.	✓
26			The HCM shares the results of the review and evaluation with the entire company.	✓

#### 2.6.2 Identifying necessary adaptations for optimization

During structured discussions, the intervention providers and research team evaluated whether each modification documented in the checklists constituted a necessary adaptation for optimization. Seven adaptations were identified by consensus and categorized according to their corresponding core functions and forms, as shown in Table 5. Discussions also examined the acceptability of core functions, forms, and adaptations from the perspective of intervention providers. To support interpretation of adaptations, descriptive statistics on smoking abstinence rates and program acceptability were calculated. The results are presented in the Results section.

**Table 5:** Adaptations necessary for optimization of the eSMART-TC implementation strategies.

No.	Form	Core function	Adaptation and the reason for it (Module 1)	What is being modified? (Module 2)	What is the nature of the modification? (Module 3)	Reason for the adaptation (Module 4)	Reason for the adaptation (Module 4)	Was it planned or spontaneous? (Module 5)	Who made the decision (Module 6)?	How broadly the changes are made and the impact of those changes.
No.	Forms	Core Functions	*Module 1: Modifications*	*Module 2: What is modified?*	*Module 3: Nature of the modification?*	*Module 4: What is the goal?*	*Module 4b_what is the level of the rationale of the adaptation? (socio-political, organizational, provider, recipient/patient)*	*Module 5: When is the modification made*	*Module 6: Who participates in the decision to modify?*	*Module 7:How widespread is the modification?*
1	The employer participates in Sessions 1–3.	Ensuring executive engagement and support	Participation in Sessions 0–3 was generally required; however, with close collaboration between the HCM and the employer, the duration could be shortened. If requested, continued participation was acceptable. Each company strengthened executive involvement using its own approach.	Contents	Removing/skipping elements	Decrease costs of the implementation effort/Increase the acceptability, appropriateness, or feasibility of the implementation effort	Organizational level	Implementation phase/ Reactive adaptation	Employer, HCM, PHN	Organization
					Shortening/condensing					
					Lengthening/extending					
2	The second to final sessions are conducted via web and last 30–60 min.	Ongoing facilitation tailored to the workplace context	The delivery method (in person or web-based) was selected according to company preferences and circumstances, as well as supporters' comfort. Session duration ranged from 30 to 60 min.	Contents	Tailoring/tweaking/refining	Increase the acceptability, appropriateness, or feasibility of the implementation effort	Pre-implementer level	Implementation phase/ Proactive adaptation	HCM, PHN	Organization
3	Conduct 10–12 sessions over 6 months.	Ongoing facilitation tailored to the workplace context	The number of sessions could be adjusted based on company needs, time availability, and campaign content.	Contents	Tailoring/tweaking/refining	Increase the acceptability, appropriateness, or feasibility of the implementation effort	Implementer level	Implementation phase/ Reactive adaptation	HCM, PHN	Organization
4	Is a preparation period of 1 month acceptable?	Supporting employees to quit smoking through the cessation campaign	The preparation period was shortened or extended to allow companies to develop satisfactory plans and ensure sustainability.	Contents	Tailoring/tweaking/refining	Increase the acceptability, appropriateness, or feasibility of the implementation effort	Implementer level	Implementation phase/ Reactive adaptation	HCM, PHN	Organization
5	The campaign recruitment period is 1 month.	Supporting employees to quit smoking through the cessation campaign	The recruitment period was modified according to the company's busy periods and industry type, with some companies opting for shorter or longer durations.	Contents	Tailoring/tweaking/refining	Increase the reach of the EBP	Implementer level	Implementation phase/ Reactive adaptation	HCM, PHN	Organization
6	The smoking cessation challenge period is 4 months.	Supporting employees to quit smoking through the cessation campaign	The challenge period was lengthened or shortened based on preferences, such as increasing participation, allowing relapsed smokers to try again, or aligning with the fiscal year.	Contents	Tailoring/tweaking/refining	Increase the acceptability, appropriateness, or feasibility of the implementation effort/Increase the reach of the EBP	Implementer level	Implementation phase/ Reactive adaptation	HCM, PHN	Organization
7	The entry method requires submission of a smoking cessation declaration.	Supporting employees to quit smoking through the cessation campaign	It was decided that submitting a smoking cessation declaration form was not necessary, and oral confirmation or submission of other documents would also be acceptable.	Contents	Tailoring/tweaking/refining	Increase the reach of the EBP	Patient or Other recipient level (employee)	Implementation phase/ Reactive adaptation	HCM, Employee, PHN	Organization

### 2.7 Qualitative analytic procedure

Qualitative analysis was conducted to define core functions and forms of the eSMART-TC implementation strategies and to identify modifications considered necessary adaptations for optimization. Data sources included: (1) implementation strategy checklists completed by intervention providers for each company (*n* = 16 checklists, one per company, documenting all sessions from initial assessment through final review), and (2) documented outputs from nine structured provider discussion sessions (three sessions per provider group).

Analysis followed a directed, framework-based approach guided by FRAME-IS and the Function and Forms Matrix. Two members of the research team (MO and JS) independently reviewed submitted checklists to identify instances in which planned implementation strategies were not delivered as specified. Each discrepancy was documented and provisionally classified according to FRAME-IS modules (action, actor, action target, temporality, dose, implementation outcome, and justification).

To enhance trustworthiness and consistency of the interpretations, multiple validation strategies were employed. First, provisional classifications were independently reviewed by two analysts (MO and JS), with discrepancies resolved through discussion and, when necessary, consultation with a third team member (TS). Second, all classifications were validated through member checking during structured provider discussions, ensuring alignment between framework-based interpretation and actual implementation experience. Third, consensus decisions were documented and reviewed across multiple sessions to ensure consistency over time and across provider groups. This multi-layered validation process ensured that the results of the analysis reflected both rigorous framework application and practical implementation realities.

This study follows the StaRI and SRQR reporting guidelines; completed checklists are provided as Supplementary Files.

## 3 Results

### 3.1 Core functions and forms of implementation strategies

Among the 26 implementation strategies (forms), a subset was identified as key forms based on patterns of modification observed during implementation (Table 4). Eight forms (No. 1, 2, 4, 5, 7, and 23–25) were consistently delivered without modification across all companies. Furthermore, 10 forms were modified in some companies but were ultimately retained as key forms because the observed modifications were either undesirable (e.g., omissions due to oversight or modifications associated with negative outcomes) or limited to highly specific contexts not generalizable to typical SME settings (e.g., companies with very low smoking prevalence or medical institutions with specialized resources). Among these modified forms, No. 3, 8, 9, 10, and 13–18 were designated as key forms following consensus discussions with intervention providers.

Through discussion with intervention providers, one additional implementation strategy—No. 26: “The HCM shares the results of the review and evaluation with the entire company”—was designated a key form due to its role in promoting internal communication and organizational learning. As a result, 19 implementation strategies were officially designated as key forms (Table 4).

The key forms of eSMART-TC's implementation strategies were organized into four core functions addressing four motivating needs and 26 forms to operationalize each function effectively. The first core function, Ensuring Executive Engagement and Support, was developed to address the challenge that limited executive commitment impedes the creation of a health-conscious organizational culture and reduces the likelihood of achieving meaningful outcomes. Six forms were assigned to this function, including setting objectives for tobacco control and issuing a formal implementation declaration to employees. The second core function, Ongoing Facilitation Tailored to the Workplace Context, aimed to mitigate the tendency for health promotion to be deprioritized amid competing business demands and to ensure facilitation throughout all phases of implementation. Forms supporting this function included conducting regular intervention sessions, requiring the HCM to submit an action memo reporting on progress between sessions, and providing facilitation based on these updates. The third core function, Supporting Employees to Quit Smoking through the Cessation Campaign, was designed to increase motivation among employees to stop smoking. HCMs were responsible for disseminating evidence-based cessation methods and delivering support tailored to employees' readiness to quit. The final core function, Review and Evaluate the Campaign, addressed the difficulty of sustaining the Plan-Do-Study-Act (PDSA) cycle over time. To maintain ongoing improvements, forms required that tobacco control efforts be reviewed during established company health meetings, such as the hygiene committee, and that new goals be set based on these evaluations. Additionally, intervention providers, particularly PHNs serving as external change agents, emphasized the importance of conducting evaluations and sharing results internally, which was adopted as an additional form through consensus discussions. Table 4 summarizes the motivating needs, core functions, and forms using the Function and Forms Matrix.

### 3.2 Adaptations identified through FRAME-IS

Adaptations essential for optimization were specified within seven forms across three core functions. Based on a review of documented modifications using FRAME-IS elements and structured discussions with intervention providers, seven such adaptations were identified by consensus and categorized according to the core function and form they supported (Table 5).

#### 3.2.1 Ensuring executive engagement and support

Modifications concerning executive engagement were implemented reactively during the implementation phase to address employer participation in sessions. Although employers were asked to attend sessions to develop implementation plans for tobacco control measures, the employers of two companies were unable to participate due to work demands. To address this barrier, the provider instructed the HCM to consult with the employer before each session and to convey the provider's guidance afterward. In company A, the employer continued to attend sessions intermittently after session 3. This employer demonstrated a high level of commitment to employee well-being and showed strong dedication to health promotion. Furthermore, the HCM, who was a smoker, decided to quit smoking as part of the campaign while supporting other employees in cessation efforts. In this company, sustained employer involvement proved beneficial. The employer was able to share the challenges experienced by the HCM, who needed to quit smoking personally, while also assisting employees with cessation.

This continued engagement enabled prompt decisions about additional support measures during intervention sessions. Consequently, the smoking cessation success rate in this company increased to 30.8%, with 4 of 13 baseline smokers successfully quitting. Discussions with providers underscored the importance of employer involvement. One provider noted, “It is essential that the HCM does not become isolated. Providing opportunities for employers to directly ask questions or seek advice from providers can be highly beneficial”. Other providers emphasized the value of employer participation, stating, “If the employer does not attend the sessions, it is difficult for the initiative to be recognized as a company-wide effort. Their involvement in setting objectives and goals is crucial,” and, “To enhance the sustainment of tobacco control measures, it is desirable for the employer to be present at the final review session as well”. Simultaneously, providers described challenges in maintaining active employer involvement and identified the need for support to secure their engagement with enthusiasm and commitment. As a result, employer participation in sessions was established as a standard practice for sessions 0–3. However, if strong collaboration between the HCM and the employer had already been established, participation could be shortened, and if the employer wished to remain engaged, involvement could continue beyond session 3. This adaptive approach—tailoring the level of executive engagement to each company's characteristics and preferences—was considered necessary to optimize the implementation strategy.

#### 3.2.2 Ongoing facilitation tailored to the workplace context

During the RCT phase, facilitation was delivered through online sessions. However, in the pre-implementation phase, discussions between providers and HCMs prompted a proactive modification to conduct an initial face-to-face session. Many intervention providers were PHNs from JHIA, who typically deliver health guidance and company support through in-person visits. They reported that, considering their work practices, they felt more comfortable with face-to-face interactions than web-based sessions. Meeting company representatives in person before initiating online support was believed to help establish a stronger foundation of trust between the intervention provider and the company. PHNs also noted that directly verifying the degree of implementation of tobacco control measures (e.g., whether a total smoking ban or a designated smoking area had been enforced) and observing employee movement patterns and structural characteristics of the workplace (such as whether the business operated in a company-owned building, rented space, or isolated house) offered significant advantages. This information was considered essential for providing effective support for tobacco control measures. As a result, both web-based and in-person sessions were adopted as adaptable forms, depending on the needs of the company and the intervention provider.

Another adaptation involved delivering 10–12 sessions during the 6-month intervention period. This modification was implemented reactively during the implementation phase after discussions between HCMs and intervention providers. In the RCT phase, a minimum of 10 sessions was required regardless of the company's progress in adopting tobacco control measures. However, some companies demonstrated high readiness for health promotion, possessed sufficient knowledge of tobacco control, and were already implementing measures with strong commitment, making 10 sessions unnecessary. Consequently, the number and frequency of intervention sessions were adapted based on the assessment of each company's challenges and specific needs.

Company B most substantially reduced the number of intervention sessions, conducting only four sessions over 6 months. The HCM had previously encouraged individual smokers to quit whenever possible but had no experience implementing company-wide tobacco control measures. To enhance acceptability, appropriateness, and feasibility, the intervention sessions primarily focused on developing an implementation plan for company-wide tobacco control measures. The smoking cessation success rate was 11.1% (1 of 9 baseline smokers successfully quit). The HCM observed a ripple effect from one employee's cessation on other smokers and expressed a strong desire to continue this tobacco control approach in the future.

#### 3.2.3 Supporting employees to quit smoking through the cessation campaign

During implementation, the company's HCM and intervention provider jointly modified the preparation period for the cessation campaign, the employee recruitment period, and the cessation challenge period. These reactive modifications aimed to improve the acceptability, appropriateness, and feasibility of implementation and to expand the reach of the EBP. The duration of each period varied depending on company readiness, seasonal workload, and whether most employees worked off-site. Intervention providers were expected to remain flexible in tailoring methods to each company's needs while maintaining ongoing facilitation. Intervention providers expressed hope that the campaign would help HCMs recognize the value of health promotion, develop effective campaign strategies, and eventually extend their efforts to broader health initiatives. One provider stated, “Our goal is for companies to independently develop and sustain their own health promotion PDSA cycles”. Thus, customizing the timing and duration of campaign components was deemed essential to optimize implementation and to support HCMs in planning with confidence.

At Company C, the preparation period lasted nearly 3 months. The HCM reported that the company broadened its focus from tobacco control measures to overall employee health promotion. The period also served as a time to reflect on the company's vision and mission, specifically its responsibility for employee well-being and the need to balance health promotion with productivity and operational efficiency. This strategic alignment allowed both the employer and HCM to fully commit to the tobacco control initiative. The resulting smoking cessation success rate was 25.0% (3 of 12 baseline smokers successfully quit). Initially, the program required employees intending to quit smoking to submit a declaration form. However, providers reported that in some companies, this formal process created psychological barriers that discouraged participation. To address this, alternative declaration methods—such as verbal notification to the HCM or sending an email—were introduced to promote inclusivity and reduce hesitation.

#### 3.2.4 Acceptability of the program, including adaptations for optimization by companies and intervention providers

During discussions with intervention providers, feedback was collected on the acceptability of core functions, key forms, and adaptations required for optimization. One provider commented, “The program targets employers and HCMs rather than individual smokers. Individual health guidance has limitations, but this approach facilitates company-wide awareness”. Another noted, “The intervention was designed to develop employers and HCMs, fostering a sense of responsibility to manage employee health”. JHIA PHNs especially valued the components Ongoing Facilitation Tailored to the Workplace Context and Supporting Employees to Quit Smoking Through the Cessation Campaign, which aligned well with their routine health guidance. Providers appreciated that the adapted program allowed companies to tailor duration and methods based on specific challenges, maintaining motivation and engagement. However, some pointed out that a flexible approach would be difficult to execute without first experiencing the default version of the program. Others noted that ensuring consistent employer involvement remained a significant challenge.

### 3.3 Intervention outcomes supporting adaptation interpretations

To support interpretation of the adaptations, descriptive statistics summarized smoking abstinence rates and program acceptability. Across 16 companies, the average smoking cessation success rate was 9.5% (range: 0%–30.8%). In the company survey, the average effectiveness rating was 4.4 points (SD: 0.51, range: 4–5), and the average satisfaction rating was also 4.4 points (SD: 0.51, range: 4–5). These results served as supplementary outcome data that supported both the appropriateness of decisions regarding whether implementation strategy adaptations were necessary in practice and the feasibility and acceptability of the adapted strategies.

## 4 Discussion

This study applied a combined FRAME-IS and Function and Forms Matrix approach to systematically examine adaptations to eSMART-TC implementation strategies during post-trial delivery. Below, we discuss the implications of our findings—including the identification of four core functions, 19 key forms, and seven necessary adaptations—for implementation strategy specification and optimization in workplace tobacco control interventions.

### 4.1 Recognition of core functions and forms

Clearly defining core functions and key forms supported fidelity while allowing context-specific flexibility. Using the Function and Forms Matrix, the eSMART-TC implementation strategy was categorized into four core functions designed to address four motivating needs or problems, along with 26 forms enforcing these functions. The adaptation frameworks FRAME–IS and MADI emphasize the importance of preserving the functionality of core functions during adaptation (15, 21). Therefore, establishing clear definitions of core functions and forms is considered a critical first step in adaptation (21). Throughout this process, fidelity was maintained in 19 of the 26 forms. Intervention providers reported that these forms contributed positively to outcomes when implemented. Accordingly, these 19 forms were identified as key forms of the implementation strategy, and it is recommended that they be implemented without modification as a general principle. However, if obstacles to implementation arise or a more effective approach is identified, planned adaptation is advised.

Previous research on workplace tobacco control has largely focused on intervention components delivered to employees rather than on implementation strategies for building organizational capacity (12). The four core functions identified in this study demonstrate both convergence with and extension of these approaches. Systematic reviews of workplace smoking cessation interventions have identified effective components, including counseling, pharmacological treatment, and smoke-free policies (22). Our “Employee cessation support” function aligns with these evidence-based elements. However, prior studies have given limited attention to the organizational implementation infrastructure required in resource-constrained settings such as SMEs (5, 6). Our three additional core functions—“Executive engagement,” “Facilitation for HCM activities,” and “Campaign design and implementation”—address this gap by building organizational capacity to deliver and sustain cessation support. Moreover, these functions correspond to strategies described in systematic reviews of workplace implementation strategies (23) and the Expert Recommendations for Implementing Change (ERIC) compilation (12), such as “develop stakeholder interrelationships,” “identify and prepare champions,” “facilitation,” and “use evaluative and iterative strategies.” These consistencies with well-established implementation strategies enhance the validity of our approach. However, our “Facilitation for HCM activities” reflects SME-specific elements not fully captured in existing frameworks: sustained, individualized support for designated healthcare managers through external public health nurses, with flexible formats and frequencies adapted to company readiness and resource constraints (5, 6). Furthermore, the systematic identification of 19 key forms advances adaptation science by operationally distinguishing essential vs. adaptable components—an issue emphasized in FRAME-IS and MADI (15, 20) but rarely specified in practice.

### 4.2 Adaptation items for optimization–emphasis on fine context-specific adjustments

Context-specific adaptations, particularly to the duration and frequency of activities, effectively improved implementation feasibility. Modifications were made to seven forms to align with each company's context and were identified as necessary adaptations for optimizing the implementation strategy. Of these adaptations, five addressed the frequency and duration of interventions. These adjustments reflected intervention providers' proposals regarding the number of sessions and time allocation based on company readiness, industry characteristics, and unique challenges. Addressing barriers such as time constraints imposed by the program and implementation challenges within JHIA also motivated these adaptations. Modifications included reducing the number of sessions during the intervention period, adjusting the time allocated to planning and campaign execution, and extending the smoking cessation campaign beyond the intervention timeframe. These changes were determined through ongoing assessments of company needs and discussions between intervention providers and company representatives. Apart from modifications to session format (in-person or online), most modifications were reactive. Although proactive modifications are generally preferred, prior research (15) indicates that flexible, adaptive responses are often necessary to address unanticipated changes in real-world settings. Reactive modifications were more common in this study for several reasons. First, because the implementation strategy spanned preparation, implementation, and maintenance phases, it was often difficult to assess the need for adjustments until each phase was underway. Second, modifications incorporated findings from needs assessments conducted by HCMs among employees during implementation. This study distinguished between “modification” (any change to implementation strategies) and “adaptation” (intentional, contextually appropriate changes to improve fit), regardless of whether changes were planned or reactive (19, 24). This distinction follows frameworks such as FRAME-IS and MADI.

Therefore, it is necessary to distinguish between planned and reactive modifications when documenting them. In this study, reactive modifications were implemented flexibly through consensus-building between intervention providers and companies to ensure the most appropriate approach in each case. Because these modifications were systematically recorded, the knowledge gained about specific contexts and necessary adjustments can be shared among intervention providers. Over time, accumulating such records can help build evidence on effective modifications for particular settings.

However, compared to planned modifications, reactive modifications require caution, as their impact can be difficult to predict, they may deviate from core functions, and they risk inconsistency (15). Modifications should never be made arbitrarily; they must maintain fidelity to core functions, as deviations can undermine the intended effectiveness of the strategy (18). Implementing proactive adaptations rather than relying solely on reactive modifications can help increase implementation success while preserving core function integrity (25). Therefore, it is essential to train intervention providers to verify fidelity to core functions when making reactive modifications. Training should also include conducting systematic needs assessments, improving the fit of the strategy to the company's context, and planning modifications to address existing constraints effectively.

### 4.3 Future implementation

Collaborative refinement during implementation supports feasibility, motivation, and sustainability. In this study, intervention providers conducted three discussions during and after the intervention to finalize the core function matrix and determine necessary adaptations for optimization. During these discussions, intervention providers expressed positive expectations for the program and noted that adaptations helped sustain the motivation of participating companies. In future studies, combining FRAME-IS with a tracking framework such as the Longitudinal Implementation Strategy Tracking System (LISTS) may further enhance the ability to capture how implementation strategies are applied and evolve over time (26).

Although adaptations aim to improve processes, if forced or introduced hastily, they may compromise service delivery and effectiveness by reducing feasibility and fidelity (27). Modifications to implementation strategies align with a co-creation model, in which researchers and stakeholders—including implementers, policymakers, and community members—collaborate to identify needs, generate knowledge, and adapt strategies to local contexts. This approach emphasizes equal partnerships, shared decision-making, and mutual learning, and has been applied across health, welfare, and community-based research settings (28–30). Within this framework, contextual adaptation is not only expected but essential for sustainable and acceptable implementation (20). Moreover, adapting strategies through co-creation with frontline healthcare providers, patients, and policymakers has been reported to improve acceptability and enhance sustainment in practice (31). Therefore, rather than delivering standardized programs or implementation strategies, adopting a flexible approach consistent with the co-creation model will be crucial for achieving effective and lasting implementation.

### 4.4 Strengths

This study offers a new perspective and framework for advancing fidelity-aligned yet adaptive implementation. It has several strengths, including the real-time documentation of modifications, theoretical systematization of core functions and forms, addressing implementation barriers, and application of the co-creation model. By integrating these elements, this study goes beyond merely recording modifications and provides a novel perspective on optimizing and sustaining implementation strategies, contributing significantly to the academic field. Furthermore, the proposal of key forms represents a major strength. While previous research has extensively discussed the adaptation of implementation strategies, systematic efforts to identify which forms are essential for successful implementation have been limited (15, 21). In this study, by applying the core function and forms framework, 19 of 26 forms were identified as key forms recommended for implementation without modification in principle. This approach offers a new guideline for balancing fidelity and adaptation and has substantial practical value.

### 4.5 Limitations

This study has several limitations. First is the limited number of participating companies and the difficulty in evaluating the impact of adaptations. Because adaptations were made continuously and tailored to each company's context, variations arose in the implementation process and approach, making it impossible to directly compare the effects of interventions with and without adaptations under equivalent conditions.

Ideally, a study design comparing groups with and without adaptations across multiple companies sharing similar contexts—such as industry type, size, and employer leadership—would have enabled a more rigorous evaluation of adaptation effects. Future research should document adaptation processes in greater detail and employ impact-oriented frameworks such as MADI (21) to assess adaptation effects more clearly.

The second limitation is the lack of quantitative measurement of intervention providers' evaluation and acceptability of the program. This study did not conduct quantitative assessments of how intervention providers perceived and accepted the program. Consequently, it was not possible to quantify the extent to which the adapted program was accepted or to identify factors influencing implementation and outcomes based on systematic data. Although qualitative data, including interviews and feedback, were collected, combining them with quantitative measures would allow for a more robust assessment of providers' perspectives. Future research should incorporate quantitative evaluation methods to clarify how factors such as program acceptability, feasibility, and sustainment at the provider level affect implementation success. Moreover, the designation of key forms is inherently context dependent. The determination that specific forms are essential for maintaining fidelity was based on implementation experiences within Japanese SME settings and should not be generalized to other organizational contexts, cultural settings, or healthcare systems. As implementation data accumulate across more diverse settings, the classification of forms as “key” vs. “adaptable” may need to be revisited. Future research should examine whether these key forms remain critical across varied contexts or whether different forms emerge as essential in other implementation environments.

## 5 Conclusion

In this study, FRAME-IS was applied to record and analyze adaptations within the eSMART-TC implementation strategy, and the core functions and forms were organized. Among the 26 forms, seven were identified as requiring adaptation to optimize the implementation strategy. While many adaptations were implemented reactively, they were executed appropriately through discussions with companies and providers.

Additionally, the use of FRAME-IS enabled detailed documentation of adaptations and generated insights that contribute to the evaluation and improvement of implementation strategies. Planned adaptations and training focused on maintaining consistency with core functions will be important in future implementation.

In particular, the proposal of 19 key forms based on the core function and forms framework offers a practical guideline for balancing fidelity and adaptation. This contribution provides not only theoretical value but also actionable direction for future implementation efforts.

## Data Availability

The original contributions presented in the study are included in the article/Supplementary Material, further inquiries can be directed to the corresponding authors.
