# Supplementary material for: Adapting a workplace tobacco control program in small and medium-sized enterprises: identifying “key forms” for fidelity and flexibility using FRAME-IS
Source: Front Health Serv. 2026 Feb 11;6:1730791. doi: 10.3389/frhs.2026.1730791 (PMC12932559; doi:10.3389/frhs.2026.1730791)
Supplement: Supplementary file 2 [file Table2.docx]

Standards for Reporting Qualitative Research (SRQR)

| SRQR Item | Section(s) |
| --- | --- |
| Title and abstract reflect the qualitative nature of the study | Title, Abstract |
| Problem formulation includes a clear rationale and significance | Background |
| Purpose or research question is clearly stated | Objective |
| Qualitative approach and research paradigm identified (e.g., thematic analysis, inductive reasoning) | Methods – Data analysis |
| Researcher characteristics and reflexivity are described | Discussion – Limitations |
| Context and setting of the study are described | Methods – Study setting, Background |
| Sampling strategy is explained, including rationale for sample size | Methods – Participants |
| Ethical issues including approval and consent are addressed | Declarations – Ethics approval |
| Data collection methods are clearly described and justified | Methods – Data collection |
| Instruments and technologies used are described (e.g., interview guides, checklists) | Methods – Data collection |
| Units of study are specified (e.g., participants, organizations) | Methods – Participants |
| Data processing methods are detailed (e.g., transcription, coding) | Methods – Data analysis |
| Data analysis methods are described, including who was involved and how | Methods – Data analysis |
| Techniques to enhance trustworthiness are described (e.g., triangulation, member checking) | Methods – Data analysis |
| Synthesis and interpretation of results are clearly presented | Results – Core Functions and Forms / Adaptations Identified |
| Links to empirical data (e.g., quotes, examples) are included | Results – Adaptations Identified |
| Integration with prior work and implications discussed | Discussion |
| Limitations of the study are acknowledged | Discussion – Limitations |
| Conflicts of interest and funding sources are disclosed | Declarations – Competing interests |
| Funding | Declarations – Funding |
